# Supplementary figures and images for: How the dynamic interplay of cortico-basal ganglia-thalamic pathways shapes the time course of deliberation and commitment
Source: PLoS Comput Biol. 2026 Mar 9;22(3):e1012966. doi: 10.1371/journal.pcbi.1012966 (PMC12995308; doi:10.1371/journal.pcbi.1012966)

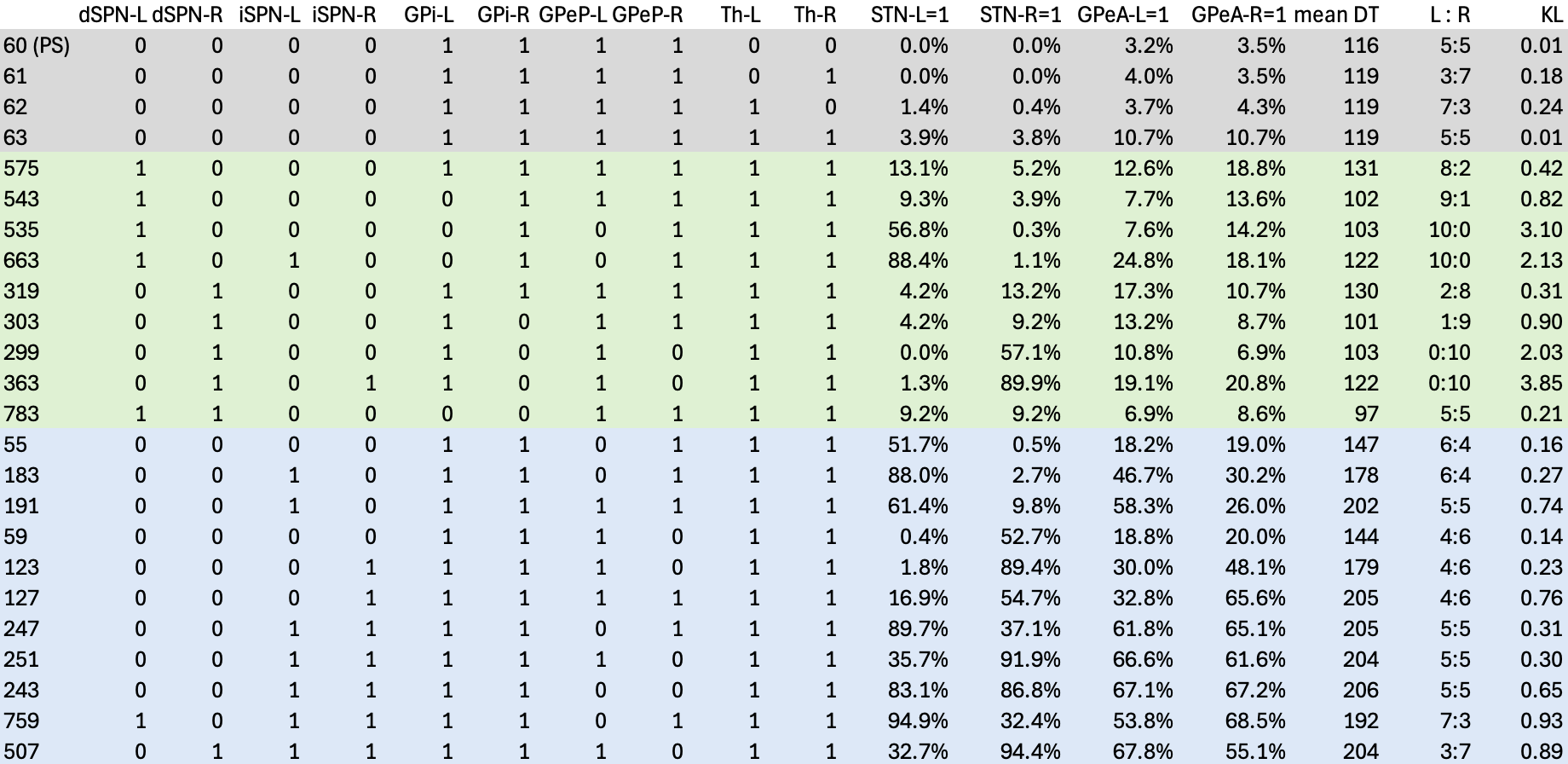

Supplement: S1 Table — The table in Fig 3 includes the details of the left-related and neutral states in the upper half of the CLAW, while this table provides the information for all CLAW states, with each pair of left- and right-related states showing symmetry up to the swap of certain L and R channel binary values. (PNG) [file pcbi.1012966.s001.png]

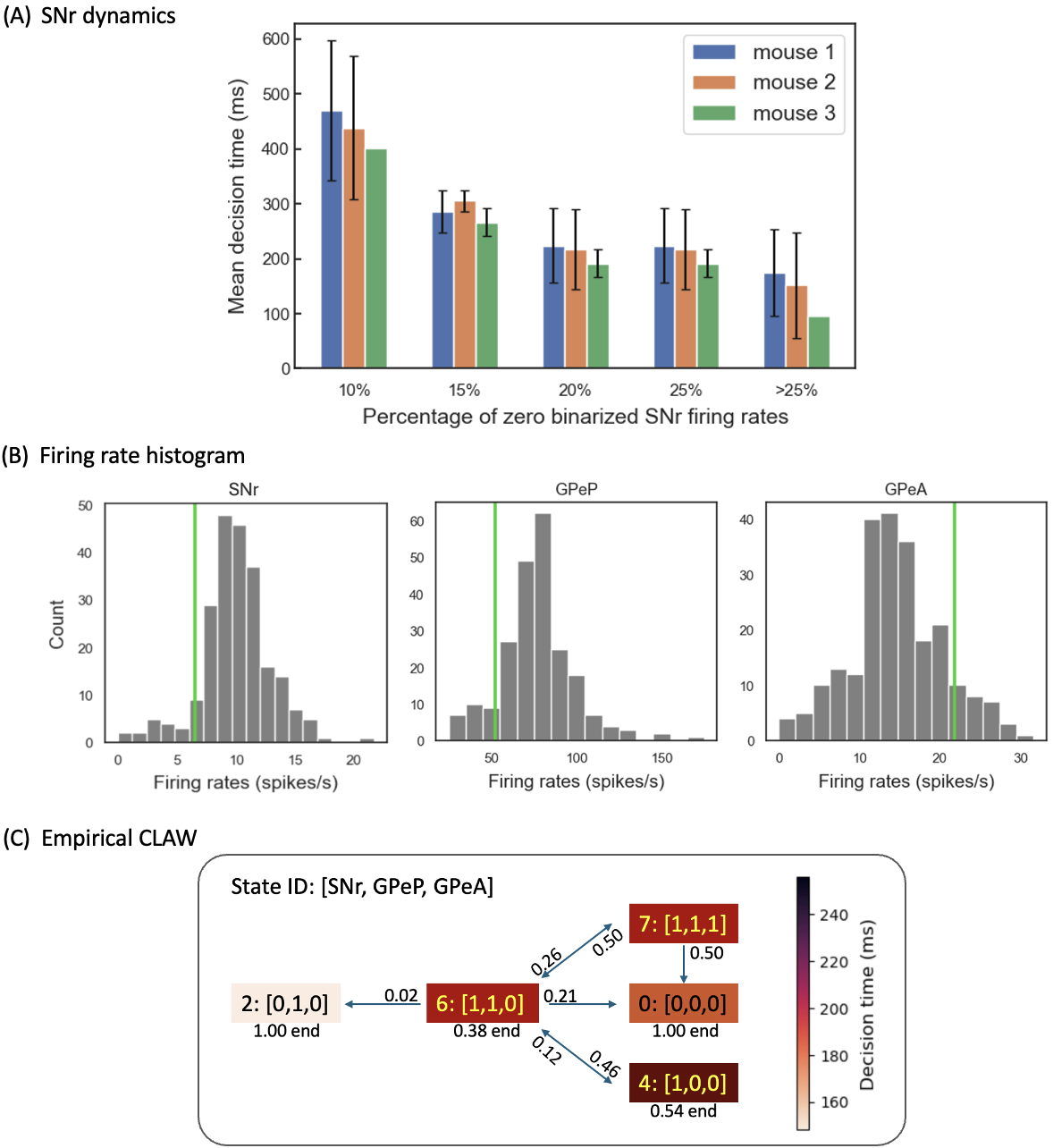

Supplement: S1 Fig — (A) The effect of SNr suppression on decision times (DTs). Bars represent the mean DT (± standard error) across trials, grouped by the percentage of zero binarized SNr firing rates during the decision time. Each color corresponds to a different mouse. (B) Firing rate histograms for SNr, GPeP, and GPeA neurons from mouse 3 across all trials up to the decision times. The vertical green line indicates the binarization threshold for each population. (C) Emprical CLAW for mouse 3, built upon the states of SNr, GPeP, and GPeA binarized activity. Numbers in boxes indicate the network states (e.g., “6: [1,1,0]” denotes state 6 where the binary firing rates of SNr, GPeP, and GPeA are 1, 1, and 0, repsectively). The transition probability from a current state to a subsequent state is indicated by the number near the arrow pointing from the current state. Numbers below states (except state 7) represent the probability of reaching decision from this state. The coloring of each state box corresponds to the mean DT of all trials that visit this state. (PNG) [file pcbi.1012966.s004.png]

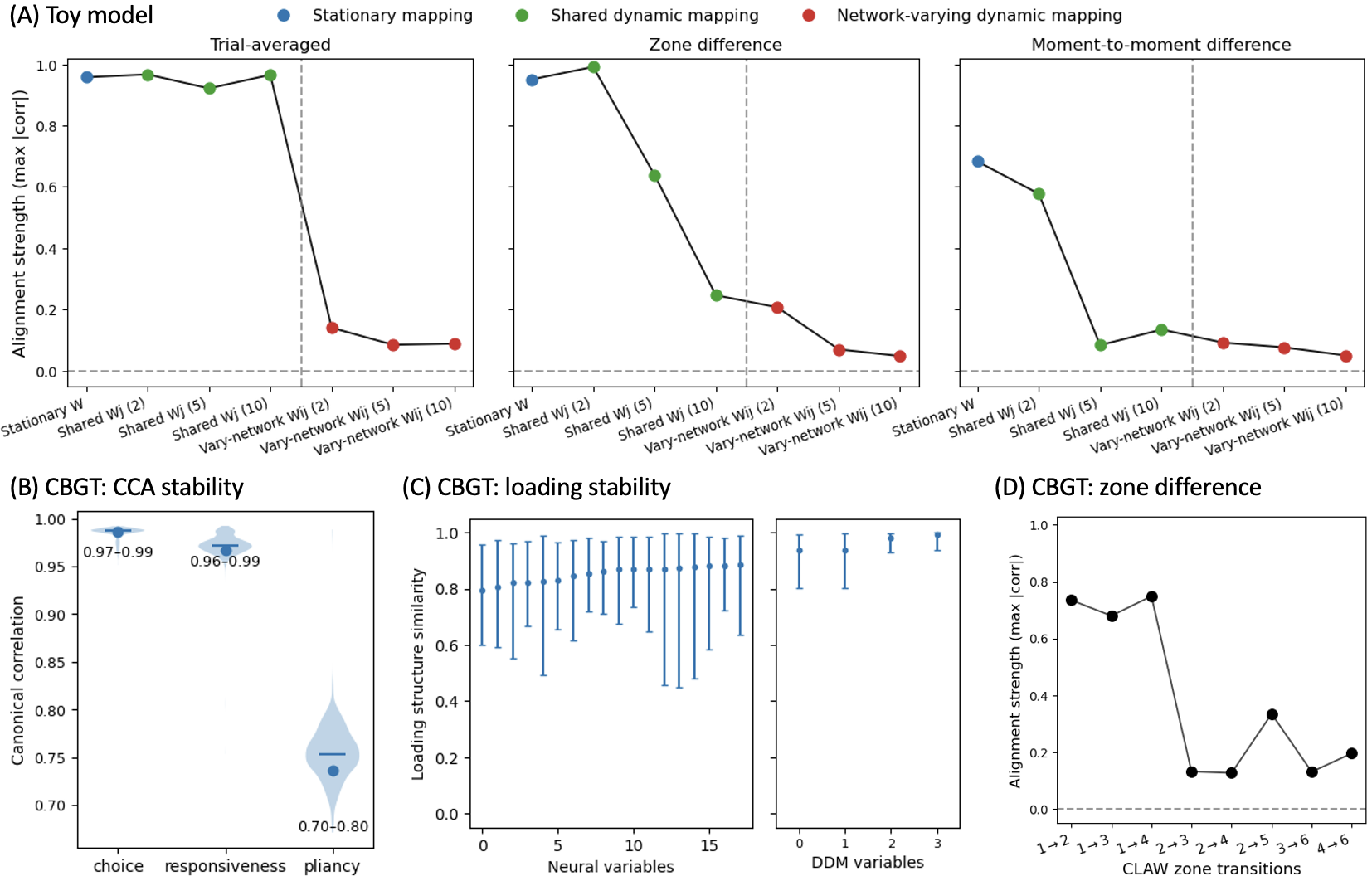

Supplement: S2 Fig — (A) Toy model: alignment strength (maximum absolute correlation across canonical dimensions) between neural and DDM projections at three temporal resolutions (trial-averaged, zone/bin differences, and moment-to-moment differences) under stationary (blue), shared dynamic (green; 2/5/10 segments), and network-varying dynamic (red) mappings. Static CCA axes were fit on trial-averaged data and evaluated on held-out networks. Alignment is preserved for stationary and shared mappings at coarse scales, degrades at finer scales, and collapses for network-varying mappings. (B–C) CBGT bootstrap stability: network-level bootstrap refits of static CCA showing stable canonical correlations (panel B), and per-variable loading structures for neural and DDM variables (panel C) with 95% intervals. (D) CBGT zone differences: the toy-model train/test alignment analysis applied to CLAW zone-to-zone activity differences using static CCA axes learned from trial-averaged data. (PNG) [file pcbi.1012966.s005.png]
