# Supplementary material for: How the dynamic interplay of cortico-basal ganglia-thalamic pathways shapes the time course of deliberation and commitment
Source: PLoS Comput Biol. 2026 Mar 9;22(3):e1012966. doi: 10.1371/journal.pcbi.1012966 (PMC12995308; doi:10.1371/journal.pcbi.1012966)
Supplement: S1 Appendix — To compare the CBGT dynamics predicted by our model with real brain dynamics, we applied our CLAW framework to the neural recording data from [87]. Our observations (see S1 Fig) demonstrate that the dynamics predicted by our theoretical CLAW—such as the relationship between SNr/GPi suppression and decision speed, and the distinct influence of CBGT pathways on the decision course—are also reflected in large-scale neural recordings, supporting the potential of our framework for interpreting the dynamics of real brain decision circuits. (PDF) [file pcbi.1012966.s002.pdf]

**S2 Appendix.** **Applying CLAW to neural recordings.** To compare the CBGT dynamics predicted by our model with real brain dynamics, we applied our CLAW framework to the neural recording data from Steinmetz et al. (2019). First, we focused on recordings from the substantia nigra pars reticula (SNr)—as a proxy for GPi—in four mice with SNr activity recorded. One mouse was excluded from analysis because its SNr firing rates fell below the range reported in the experimental literature. For each of the other three mice, we binned and binarized the SNr firing rate time series the same way as we did for our simulated data. Then for each trial, we calculated the percentage of time bins in which the binarized SNr firing rate was zero over the course of the decision time and grouped all trials into five classes based on this percentage (S1 Figure A). We observed that trials with a lower percentage of zero binarized SNr firing rates exhibited longer decision times. This result is consistent with the GPi dynamics captured by our CLAW, where prolonged GPi/SNr activity above threshold is associated with slower decisions (inner CLAW; Fig 3AB) than those where GPi/SNr dips below threshold along the way (outer CLAW).

Second, we focused on mouse 3, the only mouse with both SNr and GPe activity simultaneously recorded. Although the dataset did not specify GPe neuron subtypes, we inferred putative cell types based on neuronal firing rates: neurons with resting firing rates below 20 Hz were classified as arkypallidal, and those above this threshold as prototypic (Dodson et al., 2015). In this way, we could approximate the direct pathway dynamics from SNr activity, the indirect pathway from GPeP, and the pallidostriatal pathway from GPeA. Using firing rate histograms of the three populations (S1 Figure B), we constructed the empirical CLAW for mouse 3, as shown in S1 Figure C. As expected from our computational analysis, all trajectories originated from state 6, where SNr and GPeP were active while GPeA was inactive. We observed that trials dominated by the direct pathway were associated with the shortest decision times ($6 \to 2$, committed path) while trials dominated by the indirect pathway exhibited the longest ($6 \to 4$, deliberative path). Those with balanced activation across direct and indirect pathways showed intermediate decision times ($6 \to 0$). Here, the activation of arkypallidal neurons (state 7) also prolonged the decision process through deliberation, though the change was not significant in this case: the mean decision time of trials following $6 \to 7 \to \cdots \to 0$ was 213 ms, compared to 207 ms of trials following $6 \to 0$. Together, these observations demonstrate that the dynamics predicted by our theoretical CLAW—such as the relationship between SNr/GPi suppression and decision speed, and the distinct influence of CBGT pathways on the decision course—are also reflected in large-scale neural recordings, supporting the potential of our framework for interpreting the dynamics of real brain decision circuits.

# References

Dodson, P. D., Larvin, J. T., Duffell, J. M., Garas, F. N., Doig, N. M., Kessaris, N., Duguid, I. C., Bogacz, R., Butt, S. J., and Magill, P. J. (2015). Distinct developmental origins manifest in the specialized encoding of movement by adult neurons of the external globus pallidus. *Neuron*, 86(2):501–513.

Steinmetz, N. A., Zatka-Haas, P., Carandini, M., and Harris, K. D. (2019). Distributed coding of choice, action and engagement across the mouse brain. *Nature*, 576(7786):266–273.
