# Supplementary material for: How the dynamic interplay of cortico-basal ganglia-thalamic pathways shapes the time course of deliberation and commitment
Source: PLoS Comput Biol. 2026 Mar 9;22(3):e1012966. doi: 10.1371/journal.pcbi.1012966 (PMC12995308; doi:10.1371/journal.pcbi.1012966)
Supplement: S2 Appendix — We evaluated whether the static CCA loadings learned from trial-averaged activity (Fig 6) can support inference about dynamic decision policy changes (Figs 7–9), using a toy-model generalization-and-alignment test. In the toy model, static CCA axes generalized to coarse temporal changes when the underlying neural–policy mapping was stationary or slowly time-varying and shared across networks, but failed when the mapping was network-specific (panel A in S2 Fig). Consistent with the toy model results, our CBGT simulated data showed tight bootstrap intervals for canonical correlations and stable per-variable loading structures, and exhibited strong alignment for CLAW zone transitions (panels B–D in S2 Fig), supporting a shared neural–policy mapping across networks. Hence, our analysis of using the static CCA subspace to interpret bin/zone-scale dynamics is justified at the temporal scale relevant here (≥10 ms), even though it is not expected to capture moment-to-moment (≤1 ms) fluctuations. (PDF) [file pcbi.1012966.s003.pdf]

S2 Appendix. Validation of static-to-dynamic neural–policy mapping. To evaluate whether static CCA loadings learned from trial-averaged data (Fig 6) can support inference about time-resolved (dynamic) DDM parameters (Figs 7–9), we implemented a toy model simulation in which neural population activity $\mathbf{f}(t) \in \mathbb{R}^N$ was generated from DDM variables $\mathbf{d}(t) \in \mathbb{R}^4$ according to

$$\mathbf{f}(t) = W(t)\mathbf{d}(t) + \epsilon(t),$$

where $\epsilon(t)$ denotes Gaussian white noise. We considered two regimes of neural-policy mapping. **(A) Stationary mapping**: $W(t) \equiv W$, where the same linear mapping applied across time and across networks. **(B) Non-stationary mapping**, where the mapping $W(t)$ changed piecewise over time. For regime B, we further distinguished two cases: **(B1)** $W(t)$ **shared across networks** and **(B2)** $W(t)$ **varying across networks**. The core idea was to fit a static CCA using trial-averaged data from a training subset of networks, yielding canonical loading pairs $(\mathbf{u}_i, \mathbf{v}_i)$. Then for held-out networks, we applied the learned canonical axes at three temporal resolutions: (i) trial-averaged (sanity check), (ii) zone/bin differences ($\geq$10 ms; the scale relevant to Figs 7–9), and (iii) moment-to-moment differences ($\leq$1 ms; the hardest case). For example, for zone-to-zone transitions, the resulting neural firing and decision policy canonical scores were respectively given by

$$s_{i,k,z}^{(f)} = \mathbf{u}_i^\top \Delta\mathbf{f}_{k,z}, \qquad s_{i,k,z}^{(d)} = \mathbf{v}_i^\top \Delta\mathbf{d}_{k,z},$$

where i, k, and z index the canonical component, network, and zone, respectively. We then assessed their alignment using a rotation-invariant metric: for each canonical dimension i, we computed the Pearson correlation between neural firing and decision policy canonical scores across held-out networks, and summarized performance by the best-aligned canonical dimension, i.e.,

$$\mathcal{A}(z) = \max_i \left| \mathrm{corr}_k \big(s_{i,k,z}^{(f)},\, s_{i,k,z}^{(d)} \big) \right|.$$

This metric directly tests whether the static neural-policy axes generalize to dynamic changes.

The results of this analysis are shown in panel A of the Supporting Information S2 Figure. In the left subpanel (trial-averaged activity), alignment was near-maximal under both the stationary mapping (blue) and the shared dynamic mapping (green), indicating that the static CCA axes were robust in these regimes. In contrast, alignment was low under the network-varying dynamic mapping (red), reflecting that no single low-dimensional mapping could generalize across networks when the mapping itself was network-specific. In the middle subpanel (zone/bin differences), alignment remained high for the stationary mapping and for the shared dynamic mapping with coarse temporal scale but decreased substantially at finer scale and collapsed when mappings varied across networks. Hence, the static CCA loadings could still generalize to dynamic fluctuations only when the underlying neural–policy relationship was shared across networks and varied moderately over time. In the right subpanel (moment-to-moment differences), alignment decreased further even under stationary and shared dynamic mappings, consistent with the increasing dominance of transient fluctuations. From this toy model, we clarify that using static CCA loadings to infer dynamic DDM parameters is justified under the regime where the underlying neural-policy mapping is stationary or shared across networks and slowly time-varying, but not when the mapping becomes network-specific. In the former regime, the static axes are sufficiently stable to remain informative at the temporal scale of interest (e.g., zone/bin dynamics) but not necessarily at the moment-to-moment level.

This leads to the key question for our CBGT simulation: which of these regimes did the underlying neural–policy mapping fall into? As a first step toward applying the toy-model generalization logic to our simulated CBGT data, we assessed the reliability of the static CCA mapping itself. Specifically, using network-level bootstrap resampling of the 300 simulated CBGT networks, we repeatedly refit the static

CCA and examined the resulting distributions of canonical correlations (panel B of S2 Figure) and loading structures (panel C). The canonical correlations were highly reproducible across bootstrap replicates: the choice component correlation remained near maximal, the responsiveness component was similarly stable, and the pliancy component was somewhat less consistent but still strongly correlated, indicating that the overall strength of the static neural–policy relationship is reliable. We then evaluated whether each individual variable preserves its loading structure between CCA components across refits. For each neural variable and each DDM parameter, we quantified its component-loading profile by taking the squared CCA loadings across the three components (making the measure sign-invariant) and normalizing them within each variable to sum to one, yielding the fraction of that variable's loading magnitude assigned to each component. Because CCA components can vary across bootstrap refits, we compared profiles in an order-insensitive manner by sorting these three fractions within each variable. Loading-structure stability was then quantified as a bounded similarity score based on the L^1 distance between the bootstrap and full-data profiles (1 = identical profile), and summarized across bootstrap resamples for each variable (panel C). We observed that the DDM parameters showed tightly preserved loading structure across resamples (median stability > 0.9). Neural variables showed more variability but were nonetheless also quite stable (median stability ≥ 0.8). This pattern reflects the fact that in our CBGT simulations, multiple neural populations contribute at comparable levels (Fig 6A), so small changes in the fitted CCA basis can shift how a given population's loading is partitioned across the three components. Importantly, these shifts occur within the same low-dimensional subspace, rather than reflecting a different mapping across bootstrap refits. Together, the tight canonical-correlation intervals and stable per-variable loading structures support the existence of a shared low-dimensional neural–policy mapping across networks and argue against a network-specific regime (illustrated by the red case in the left subpanel of panel A), in which both the correlation and loading structure would be expected to fluctuate markedly across bootstrap fits.

Having established that the static CCA in our CBGT simulation is reliably estimated and shared across networks, we finally applied the same train/test generalization approach and alignment metric used in the toy model to the CLAW zone-to-zone differences. However, in the CBGT simulations, DDM parameters were available only as static per-network fits derived from choices and decision times, so time-resolved DDM trajectories were not available and could not be used to serve as ground truth. Nevertheless, the toy model shows that the primary issue for validating static-to-dynamic inference is whether the neural–policy mapping $W(t)$ is shared or varies across networks, and we therefore tested a weaker, mapping-focused condition. The neural activity in zone or time bin z can be expressed as

$$\mathbf{f}_k(z) = W(z)\mathbf{d}_k^{\text{static}} + \mathbf{b}_k(z),$$

where $\mathbf{b}_k$ captures zone/bin-dependent effects not explained by the static DDM parameters (e.g., baseline offsets, latent network-specific state, or other unmodeled dynamics). Taking differences yields

$$\Delta\mathbf{f}_{k,z_1\to z_2} = (W(z_2) - W(z_1))\mathbf{d}_k^{\text{static}} + \Delta\mathbf{b}_{k,z_1\to z_2}.$$

Under this formulation, we can apply static loadings learned from trial-averaged data of training networks to held-out networks by computing neural and policy scores,

$$s_{i,k,z}^{(f)} = \mathbf{u}_i^\top \Delta\mathbf{f}_{k,z_1\to z_2}, \qquad s_{i,k,z}^{(d)} = \mathbf{v}_i^\top \mathbf{d}_k^{\text{static}}$$

and evaluating their alignment strength $\mathcal{A}(z)$.

Applying this analysis to the simulated CBGT data (panel D of S2 Figure), we found that alignment was high for early CLAW zone transitions and decreased for later transitions. This graded pattern again rules out a

network-specific mapping (cf. panel A, red), which would yield uniformly weak alignment, and it also rules out a strictly stationary mapping (cf. panel A, blue), which would yield uniformly high alignment. Instead, the observed pattern falls in the regime of a shared but phase-dependent mapping (cf. panel A, green). In the CBGT simulations, early zones (e.g., launching and initial deliberation) spanned a larger proportion of the decision process and therefore more closely reflected the trial-averaged neural–policy relationship captured by the static CCA axes, leading to stronger alignment. By contrast, later zones (e.g., commitment, reversal, and second deliberation) were more strongly shaped by transient fluctuations, i.e., larger contributions from the $\Delta\mathbf{b}_{k,z}$ term, that weakened the alignment. Note that the alignment strength in the middle subpanel of A was computed by pooling across all zone/bin transitions, whereas here we plot the alignment separately for each CLAW zone transition. On average, the overall alignment here was large and positive across all zone transitions, indicating that the underlying mapping did not fall into the network-varying regime, identified in the toy model analysis, in which static axes are invalid for dynamic inference. Thus, the above analysis supports our claim that in our CBGT simulations, the static CCA loadings provide a valid low-dimensional subspace for inferring drives for dynamic decision policies where the temporal scale of interest was relatively coarse ($\geq$10 ms), even though they are not expected to capture moment-to-moment ($\leq$1 ms) fluctuations.
