# Supplementary material for: How the dynamic interplay of cortico-basal ganglia-thalamic pathways shapes the time course of deliberation and commitment
Source: PLoS Comput Biol. 2026 Mar 9;22(3):e1012966. doi: 10.1371/journal.pcbi.1012966 (PMC12995308; doi:10.1371/journal.pcbi.1012966)
Supplement: Attachment — Submitted filename: rebuttal.pdf [file pcbi.1012966.s006.pdf]

We thank the referees for their careful reading and thoughtful critiques of our submission. We have revised the manuscript in light of their suggestions (*italic text*). Below we provide a point-by-point discussion of the questions raised by the referees. In the manuscript, we have marked new and revised text in red font.

1 Reviewer #1

This manuscript presents an interesting method of analysing the dynamics of neural activity during choice processes. This method is applied to an extended version of a previously published model of brain circuits involved in decision making, and reveals different possible trajectories of neural activity in the model during choice trials. The manuscript is clearly written.

Response: We thank the referee for the overall assessment of the paper. We have revised the manuscript in light of the referee's suggestions, which we discuss point-by-point below.

1.1 Major

The manuscript shows that the proposed method can be applied to analysis of simulated neural activity in a computational model, and provides insights into how this model behaves. However, for this work to have a broader impact, it would be useful to also show if the proposed method can be applied to analysing neural activity of biological neurons, and to investigate if the real brain decision circuits behave in a way predicted by the model. I feel these questions can be addressed thanks to availability of large datasets on neural activity obtained in choice tasks. For example, data from an influential study (Steinmetz, N.A., Zatka-Haas, P., Carandini, M. et al. Distributed coding of choice, action and engagement across the mouse brain. Nature 576, 266–273 (2019)) is freely available online at `https://figshare.com/articles/dataset/Distributed_coding_of_choice_action_and_engagement_across_the_mouse_brain/9974357?file=30626811`*. This study recorded activity "from approximately 30,000 neurons in 42 brain regions" including the regions simulated in the manuscript, during a choice task similar to that simulated in the manuscript. Therefore, I feel it would be insightful to test if the proposed method can be applied to such experimental data. It would be particularly useful to select recording sessions in which inserted probes crossed the regions included in the model. It would be interesting to also compare the dynamics revealed by analysis of the data with the dynamics of the model. I realize that some of the analyses of the model cannot be replicated in the data (e.g. it is not possible to distinguish if striatal recorded neurons are part of direct or indirect pathways), but even comparing dynamics across different parts of cortico-basal-ganglia-thalamic loop would be exciting.*

Response: We thank the referee for the thoughtful consideration of our computational method, and for raising a helpful suggestion to broaden its impact. Although approximately 30,000 neurons from 42 brain regions were recorded in Steinmetz et al. (2019), we note several challenges for a full comparison with our study:

- Only three brain regions in their dataset—SNr (as a proxy for GPi), CP (striatum), and GPe—were related to our CBGT network.

- The dataset did not distinguish anything analogous to action channels; striatal neurons were not classified into direct or indirect pathways; GPe neurons were not differentiated into prototypic or arkypallidal neurons.
- Probe coverage varied across the ten mice studied. For example, SNr was recorded in four mice, GPe in three mice, and only one mouse had both SNr and GPe simultaneously recorded.

We should point out that these challenges underscore both the central difficulty in using prior data to support our theoretical predictions and the critical advantage of our theoretical experiments: our findings highlight a specific configuration of tasks and region analysis as a target for future large-scale recording projects like that done by Steinmetz & colleagues (2019).

Despite these challenges, we proceeded with two directions of comparison between the dynamics revealed by the data and the dynamics of our model:

- **SNr-only analysis.** For each of the four mice with recorded activity in the SNr, we binarized the activity as we did with our model. Based on our CLAW analysis, we predict that trials that have GPi/SNr activity above threshold for longer times should have higher reaction times (corresponding to trajectories along the inner CLAW) than those where GPi/SNr dips below threshold along the way (corresponding to the outer CLAW).
- **SNr-GPe analysis.** For the only data set with simultaneous recording of both SNr and GPe neurons, we first classified putative prototypic and arkypallidal GPe neurons based on their distinct firing rate properties. Combining GPe and SNr recordings, we constructed an experimental analogue of our theoretical CLAW (i.e., an empirical CLAW), based on the activity of the three groups of neurons (SNr, GPeP, GPeA), without distinguishing action channels.

Since it is not possible to distinguish if striatal neurons are part of the direct or indirect pathways based solely on firing rates, we exclude them from our analysis. Based on these analyses, we have added the following text to the end of the Discussion:

> Optogenetics allows for targeted activation or inhibition of specific neuron populations or types ... Our results also predict that stimulation of GPeA neurons should yield prolonged deliberation. In addition to informing future optogenetic experiments, the CLAW analysis can be applied to large-scale neural datasets to extract important insights into real brain decision circuits (see Supporting Information S1 Appendix for the application to neural recording data of Steinmetz et al. (2019)). Finally, a novel prediction of our control ensemble analysis (Fig 7), in light of the composition of the responsiveness and pliancy ensembles (Fig 6), is that ...

And, we have added the following details to the Supporting Information S1 Appendix, along with a new S1 Figure. Please note that S1 Figure and Fig 3 mentioned in the main text, are also shown as Figures 1 (page 4) and 2 (page 9) below, for reference.

S1 Appendix. Applying CLAW to neural recordings. To compare the CBGT dynamics predicted by our model with real brain dynamics, we applied our CLAW framework to the neural recording data from Steinmetz et al. (2019). First, we focused on recordings from the substantia nigra pars reticula (SNr)—as a proxy for GPi—in four mice with SNr activity recorded. One mouse was excluded from analysis because its SNr firing rates fell below the range reported in the experimental literature. For each of the other three mice, we binned and binarized the SNr firing rate time series the same way as we did for our simulated data. Then for each trial, we calculated the percentage of time bins in which the binarized SNr firing rate was zero over the course of the decision time and grouped all trials into five classes based on this percentage (S1 Figure A). We observed that trials with a lower percentage of zero binarized SNr firing rates exhibited longer decision times. This result is consistent with the GPi dynamics captured by our CLAW, where prolonged GPi/SNr activity above threshold is associated with slower decisions (inner CLAW; Fig 3AB) than those where GPi/SNr dips below threshold along the way (outer CLAW).

Second, we focused on mouse 3, the only mouse with both SNr and GPe activity simultaneously recorded. Although the dataset did not specify GPe neuron subtypes, we inferred putative cell types based on neuronal firing rates: neurons with resting firing rates below 20 Hz were classified as arkypallidal, and those above this threshold as prototypic (Dodson et al., 2015). In this way, we could approximate the direct pathway dynamics from SNr activity, the indirect pathway from GPeP, and the pallidostriatal pathway from GPeA. Using firing rate histograms of the three populations (S1 Figure B), we constructed the empirical CLAW for mouse 3, as shown in S1 Figure C. As expected from our computational analysis, all trajectories originated from state 6, where SNr and GPeP were active while GPeA was inactive. We observed that trials dominated by the direct pathway were associated with the shortest decision times ($6 \to 2$, committed path) while trials dominated by the indirect pathway exhibited the longest ($6 \to 4$, deliberative path). Those with balanced activation across direct and indirect pathways showed intermediate decision times ($6 \to 0$). Here, the activation of arkypallidal neurons (state 7) also prolonged the decision process through deliberation, though the change was not significant in this case: the mean decision time of trials following $6 \to 7 \to \cdots \to 0$ was 213 ms, compared to 207 ms of trials following $6 \to 0$. Together, these observations demonstrate that the dynamics predicted by our theoretical CLAW—such as the relationship between SNr/GPi suppression and decision speed, and the distinct influence of CBGT pathways on the decision course—are also reflected in large-scale neural recordings, supporting the potential of our framework for interpreting the dynamics of real brain decision circuits.

1.2 Minor

L.836: "path exhibited a gap of 25%" – I do not understand what it means. Could you please clarify?

Response: We thank the referee for pointing out this ambiguity. A "gap of 25%" refers to a substantial drop in the probability of transition to the next possible states—for example, 30% probability of transitioning to state i versus 5% probability of transitioning to state j ($i \neq j$)—which signals a cutoff. In the revised manuscript, we have clarified the text as follows:

> ... Specifically, for each state s_k, we sorted the subsequent n ($n < 2^N$) paths from largest

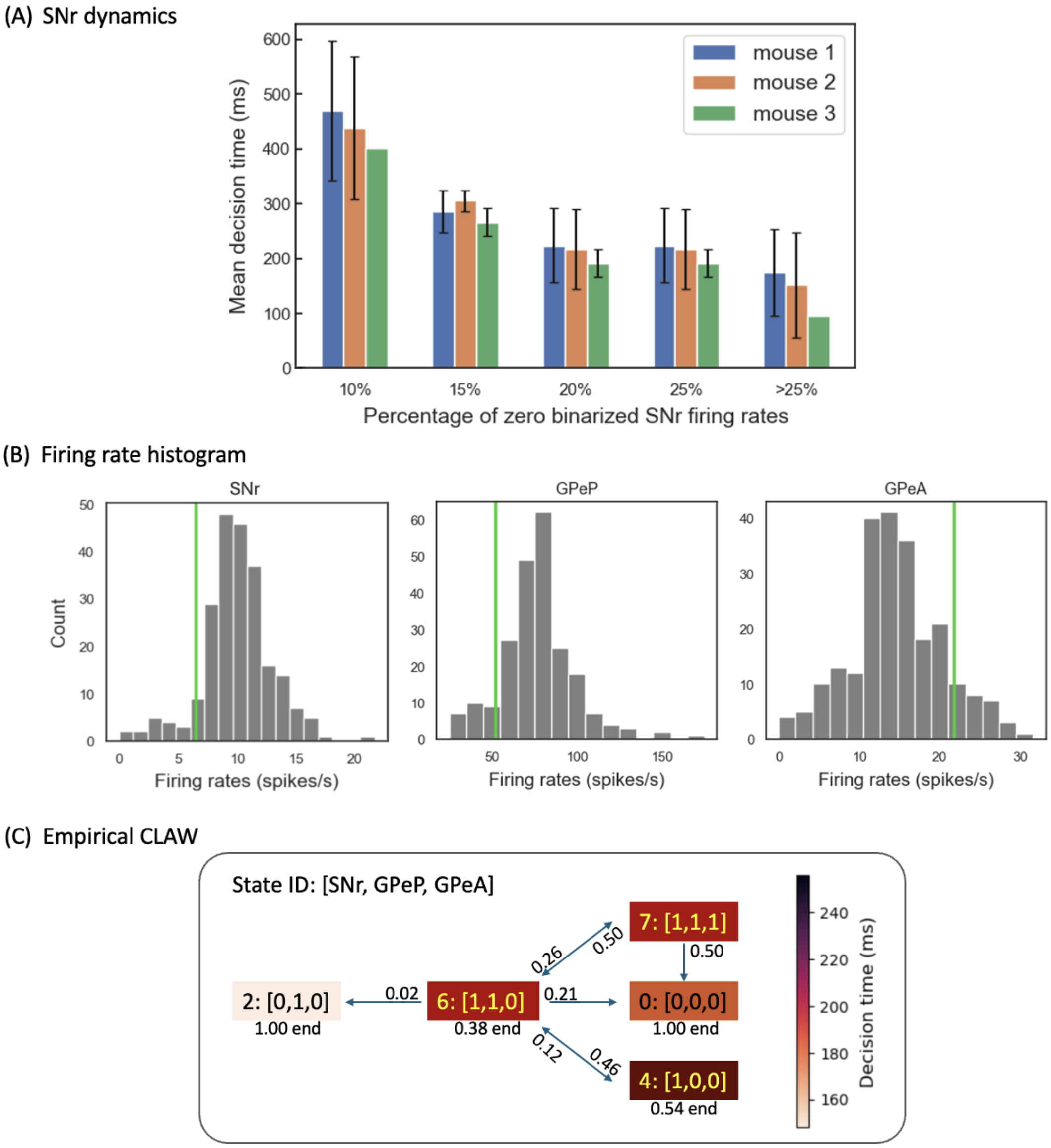

Figure 1: **Empirical CLAW of Steinmetz et al. (2019).** (A) The effect of SNr suppression on decision times (DTs). Bars represent the mean DT ($\pm$ standard error) across trials, grouped by the percentage of zero binarized SNr firing rates during the decision time. Each color corresponds to a different mouse. (B) Firing rate histograms for SNr, GPeP, and GPeA neurons from mouse 3 across all trials up to the decision times. The vertical green line indicates the binarization threshold for each population. (C) Emprical CLAW for mouse 3, built upon the states of SNr, GPeP, and GPeA binarized activity. Numbers in boxes indicate the network states (e.g., "6: [1,1,0]" denotes state 6 where the binary firing rates of SNr, GPeP, and GPeA are 1, 1, and 0, repsectively). The transition probability from a current state to a subsequent state is indicated by the number near the arrow pointing from the current state. Numbers below states (except state 7) represent the probability of reaching decision from this state. The coloring of each state box corresponds to the mean DT of all trials that visit this state.

to smallest. To focus on the most likely transitions, we retained only the top-ranked paths until a significant drop in the probability occurred—defined as a gap of more than 25% between the p-th ($p < n$) and the $(p+1)$-st paths. We treated such a gap as a cutoff, and the remaining lower-ranked paths were considered to have low likelihood and were excluded from our analysis. This whole approach for the binarization and CLAW reduces firing rate variability and provides a more refined model for the decision dynamics.

Typo: L.643: "Figs 4 8"

Response: We thank the referee for pointing this out, and we have corrected the typo:

> ... We found that, as evidence accumulates, decisions involving outer CLAW paths manifest as a strong directional preference (zones III, V, and VI; Figs 4, 8), driving the accumulation process rapidly towards a concluding outcome ...

2 Reviewer #2

In the present paper, the authors propose an innovative modeling framework to gain insights into the changes of configuration of the cortico-basal ganglia-thalamic (CBGT) network. First, they generate a variety of CBGT network models producing plausible firing rate ranges, based on predefined pools of neurons informed by the existing literature. A novel feature of their approach lies in the identification of all possible network states—defined as unique configurations of active and inactive neuronal pools—and the analysis of transition probabilities between these states. From this, they extract distinct state trajectories, notably one associated with rapid responses and another corresponding to prolonged deliberation prior to decision commitment. The authors then perform a cross-correlation analysis and replicate their previous findings, showing that variations in the global firing rates of the different neuronal pools of the models are related to behavioral decision policy along three primary dimensions: choice, responsiveness, and pliancy. These dimensions are derived from drift diffusion model (DDM) parameters fitted to the networks' behavioral outputs. Finally, they relate time-resolved firing rate variations to these previously identified decision policy dimensions and to the underlying DDM parameters. Notably, firing rate changes at state transitions are associated with collapsing decision bounds, with higher drift rates observed along the "fast response" trajectory compared to the "long deliberation" trajectory. Overall, I think this is an interesting paper, with an inventive approach for studying fine circuit-level dynamics and bridging them with the current understanding of decision processes at the computational level. However I have a significant concern (described below) regarding the computational interpretation of networks dynamics, which is in my opinion critical to address in order to include these results to the manuscript. Aside from this, I only have minor suggestions.

Response: We thank the referee for the encouraging comments and positive assessment of the paper. Below we provide a point-by-point response to the suggestions raised by the referee.

2.1 Dynamic decision policy

On page 14, starting at line 377, the authors explain that the CCA loadings relating global firing rates to three combinations of DDM parameters (i.e., decision policy dimensions or "control ensembles"), are also used to estimate dynamic decision policy variations based on within-trial firing rate variations. The authors state: "Each component of W_k represents how the instantaneous firing rate change from the $(k-1)$-th to the k-th time bin corresponds to a change in the activation, or drive, of one of the three control ensemble" (line 385-387). Later (starting on page 16, line 451), the authors apply the same kind of analysis, this time projecting firing rate changes at state transition (rather than between time bins) into the decision policy dimensions, and eventually into the space of DDM parameters. While the results presented in Figures 7 and 8 are compelling, I find the interpretation of within-trial variations in DDM parameters (or their linear combinations) to be problematic. These parameters describe distributions of behavior across trials, not time-varying variables evolving within a trial. Although some parameters, such as the boundary height or drift rate, could be interpreted as varying over time, doing so alters their original meaning, which was used to define the decision policy dimensions in the first place. For others, such as the onset time, I do not find any possible interpretation if they vary dynamically. I believe it is essential to explicit more the theoretical rationale for these analyses and to explain how within-trial changes in "control ensemble" activity should be interpreted in the context of DDM-based decision policy. Without such clarification, the computational meaning of these projections remains ambiguous. That being said, even without this computational interpretation, the identification of separate state trajectories within the CBGT network is an interesting and valuable finding in itself, especially coupled with the thorough analysis of pathways activation.

Response: We appreciate the referee's thoughtful comments about our dynamic DDM analysis. To address the referee's concern regarding the theoretical rationale of within-trial variations of DDM parameters, we have done the following:

- We have introduced more background on the classical DDM versus dynamic DDM in the second and third paragraphs of the Introduction, as indicated:

  > Prior computational work (Dunovan et al., 2019; Vich et al., 2022; Bahuguna et al., 2024) has shown how the CBGT network can regulate decision policies (e.g., manage the speed-accuracy (Schouten and Bekker, 1967; Chittka et al., 2009) and exploration-exploitation (Cohen et al., 2007; Mehlhorn et al., 2015) tradeoffs). Specifically, interactions between distinct subnetworks within the CBGT pathways can be mapped to certain behavioral outcomes described by parameters in a drift-diffusion model (DDM; Ratcliff (1978); Bogacz et al. (2006); Ratcliff and McKoon (2008))), with the low-dimensional relationship between specific configurations of CBGT networks and decision policies characterized in terms of three *control ensembles* (Vich et al., 2022). It has been further shown that dopaminergic plasticity at the corticostriatal synapses can alter the activity of CBGT control ensembles, so as to modulate the resulting decision policies in response to feedback following post-action outcomes (Bahuguna et al., 2024). The parameters of the classical DDM are typically interpreted as time-invariant quantities, estimated across *multiple* trials to capture a fixed decision policy during a decision. Thus, the previous control ensemble studies suggest that the CBGT network can establish a *static* instantiation of such a policy. In typical evidence accumulation models this would be represented by fixed values of parameters such as drift rate (the rate of evidence accumulation toward a decision) and

boundary height (the amount of evidence needed to make a decision), with gradual adaptation of these factors over the course of sequences of decisions if feedback is provided. However, it remains to be investigated how evidence accumulates through different regions of the CBGT network during the course of *individual* decisions.

A number of studies have recently challenged the assumption of static decision policies from both experimental (Cisek et al., 2009; Drugowitsch et al., 2012; Wyart et al., 2012; Thura et al., 2014) and theoretical (Krajbich et al., 2010; Huang and Rao, 2013; Moran, 2015) perspectives, showing that the classical DDM fails to capture important trends in behavioral data under some specific tasks. The intuition behind these studies is that the decision-makers adjust their decision strategies based on real-time demands. In line with this idea, there is a growing body of literature (Srivastava et al., 2017; Palestro et al., 2018; Holmes and Trueblood, 2018; Malhotra et al., 2018; Ging-Jehli et al., 2025) that explores the idea of *dynamic* evidence accumulation, in which certain parameters, particularly drift rate and boundary height, are allowed to vary within a trial to capture more realistic, temporally evolving decision policies. This raises our second question: how do the complex dynamics of CBGT networks give rise to rapid, within-trial variations in the decision policy?

- We have added the following text for Fig 7 in the main text (also see Fig. 6 below) to strenthen our explanation of how within-trial changes in control ensemble activity should be interpreted in the context of DDM-based decision policy:

  With these control ensembles in hand, we next investigated how the dynamics of CBGT network activity during decisions tunes the resulting decision policy parameters. Specifically, for each simulated trial ... Each component of W_k represents how the instantaneous firing rate change from the $(k-1)$-st to the k-th time bin corresponds to a change in the activation, or *drive*, of one of the three control ensembles. Because each control ensemble impacts specific aspects of decision policies (Fig 6B), the time series of ensemble activations imply how the CBGT network activity dynamically modulates decision policies throughout the unfolding of individual trials. Finally, we grouped all trials into four types, categorized by their decision times and selected choices: fast left, fast right, slow left, and slow right. Given that decision times vary across trials, we aligned the trials in each group to the response time, as shown in Fig 7. In this way, we converted the temporal flow of neural activity into evolving control ensemble activations, that serve as direct reflections of dynamic decision policies encoded within the course of individual decisions. For a full description of this approach, see Control ensembles in the Methods section.

- We have clarified our explanation of varying onset time for Fig 8C:

  Lastly, the onset time t consistently shortened no matter which decision path was taken. Importantly, the onset time does not vary dynamically within a trial. Instead, the effect of varying t through a CLAW path represents an updated estimate of when the evidence accumulation is presumed to begin, based on how far along the network state has progressed. For example, the drop in t associated with the progression along the inner CLAW reflected the fact that the states in zone II and zone V corresponded to being farther along in the decision process than zones I and II, respectively. When the decision trajectory reached a later stage, e.g., zone V, the process "started" faster because it had already moved past the earlier stages, i.e., zones I and II, and became more "prepared". This does not mean that the actual onset time of the trial decreases. Rather, the model adjusts the prediction for

the timing of accumulation onset as the decision process unfolds (see below and Fig 9 for more details).

2.2 Minor / Readability suggestions

Fig 3A: State IDs written in black on a dark purple background are hard to read and to find when looking for them.

Response: We thank the referee for pointing out this issue. We have revised Fig 3A in the main text (or see Fig. 2A below) by changing the text color to yellow for inner CLAW states (which have dark background) and by making all state IDs bold.

Figs 3C, 4B: These figures use the labels "D1" and "D2" to refer to D1- and D2-expressing spiny projection neurons, but the main text and the other figures all use the abbreviations"dSPN" and "iSPN", which complicates the reading.

Response: We thank the referee for pointing out this inconsistency in the usage of neuron terminology. We have revised the table labels to use "dSPN" and "iSPN" (see Figs 3C, 4B in the revised manuscript or Figs. 2B, 3B above) to match the terminology used throughout the main text and other figures.

Fig 5: Adding the zone labels (e.g. "Launching region → Left commitment") as a title for each subplot would improve readability.

Response: We thank the referee for this helpful suggestion. We have updated Fig 5 in the main text (or see Fig. 4 below) to include a title for each subplot, clarifying the corresponding zone transitions.

Fig 6B: Using the DDM full parameter names (e.g. "drift rate" instead of "v") or mentioning them in the legend would help understanding the figure independently of the main text.

Response: We thank the referee for this helpful suggestion. We have updated Fig 6B in the main text (or see Fig. 5B below) with the DDM full parameter names.

Figs 7A, 7C: The colors are very pale, making the plots quite hard to read.

Response: We thank the referee for pointing this out. We have enhanced the color contrast in Fig 7 in the main text (or see Fig. 6 below) to improve readability.

Line 48: There is a typo ("reacitve" instead of "reactive").

Response: We thank the referee for pointing this out, and we have corrected the typo:

> ... The arkypallidal neurons are currently thought to play a critical role in regulating striatal signaling, particularly during tasks requiring reactive inhibitory control ...

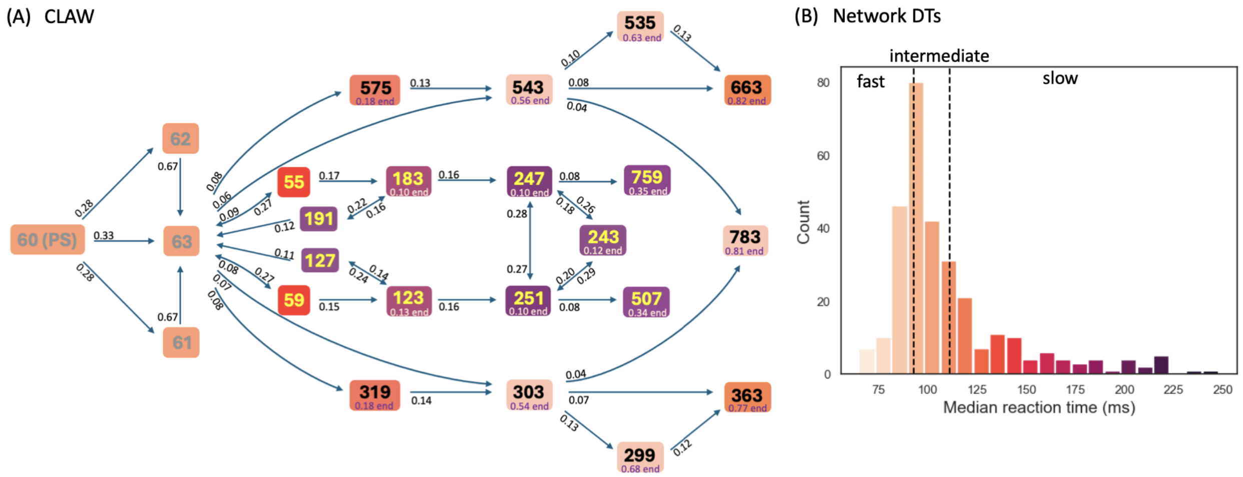

(C) State details

	dSPN-L	dSPN-R	iSPN-L	iSPN-R	GPi-L	GPi-R	GPeP-L	GPeP-R	Th-L	Th-R	STN-L=1	STN-R=1	GPeA-L=1	GPeA-R=1	mean DT	L : R	KL
60 (PS)	0	0	0	0	1	1	1	1	0	0	0.0%	0.0%	3.2%	3.5%	116	5:5	0.01
62	0	0	0	0	1	1	1	1	1	0	1.4%	0.4%	3.7%	4.3%	119	7:3	0.24
63	0	0	0	0	1	1	1	1	1	1	3.9%	3.8%	10.7%	10.7%	119	5:5	0.01
575	1	0	0	0	1	1	1	1	1	1	13.1%	5.2%	12.6%	18.8%	131	8:2	0.42
543	1	0	0	0	0	1	1	1	1	1	9.3%	3.9%	7.7%	13.6%	102	9:1	0.82
535	1	0	0	0	0	1	0	1	1	1	56.8%	0.3%	7.6%	14.2%	103	10:0	3.10
663	1	0	1	0	0	1	0	1	1	1	88.4%	1.1%	24.8%	18.1%	122	10:0	2.13
783	1	1	0	0	0	0	1	1	1	1	9.2%	9.2%	6.9%	8.6%	97	5:5	0.21
55	0	0	0	0	1	1	0	1	1	1	51.7%	0.5%	18.2%	19.0%	147	6:4	0.16
183	0	0	1	0	1	1	0	1	1	1	88.0%	2.7%	46.7%	30.2%	178	6:4	0.27
191	0	0	1	0	1	1	1	1	1	1	61.4%	9.8%	58.3%	26.0%	202	5:5	0.74
247	0	0	1	1	1	1	0	1	1	1	89.7%	37.1%	61.8%	65.1%	205	5:5	0.31
243	0	0	1	1	1	1	0	0	1	1	83.1%	86.8%	67.1%	67.2%	206	5:5	0.65
759	1	0	1	1	1	1	0	1	1	1	94.9%	32.4%	53.8%	68.5%	192	7:3	0.93

Figure 2: **CLAW (Circuit Logic Assessed via Walks) diagram for CBGT network dynamics.** (A) CLAW diagram. Bold numbers in boxes indicate the network states, and the transition probability from a current state to a subsequent state is indicated by the number near the arrow pointing from the current state. The numbers below certain states (e.g., “0.82 end” below state 663) represent the probability that if these states are reached, then they are the final states prior to decisions. (B) Overall decision time distribution across 300 networks, categorized into three equal-count tertiles defining fast (left), intermediate (middle), and slow (right) networks, demarcated by vertical dashed black lines. The coloring of each CLAW state in panel A corresponds to the mean DT for all trials that visit this state, following the same color-coding scheme as in panel B. (C) Details of the states. A complete explanation of the full set of state properties, including those related to the right choice, is presented in the Supporting Information S1 Table. From left to right, after state labels: binarized firing rates of dSPN, iSPN, GPi, GPeP, and Th for left (-L) and right (-R) channels; probability of activation (binarized firing rate = 1) for STN and GPeA for left and right channels; mean DT over the trials that visit each state; the ratio of trials that chose left/right for those that visit each state; Kullback–Leibler (KL) divergence between left and right trials’ DT distributions. The grey rows correspond to the initial states that occur early in each trial and never lead directly to a decision, and the green and blue rows correspond to outer CLAW and inner CLAW states, respectively. The states in the lower half of the CLAW are not shown; these are symmetric – up to the swap of certain L and R channel binary values – with the states that lie in corresponding positions in the upper half.

(A) Zone CLAW

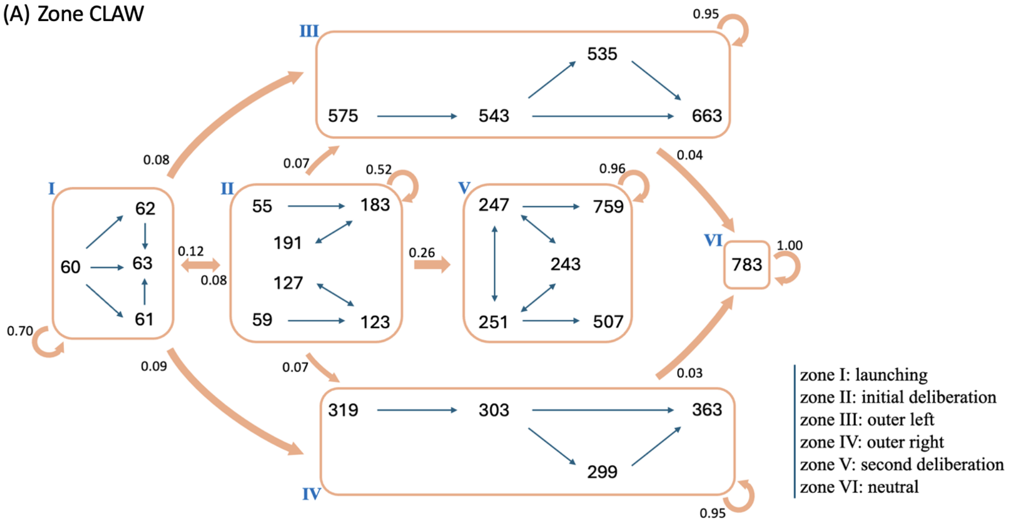

(B) Zone details

	dSPN-L	dSPN-R	iSPN-L	iSPN-R	GPi-L	GPi-R	GPeP-L	GPeP-R	Th-L	Th-R	GPeA-L	GPeA-R	STN-L	STN-R
I	0	0	0	0	1	1	1	1	0.23	0.23	0.05	0.05	0	0
II	0	0	0.25	0.26	1	1	0.57	0.57	1	1	0.30	0.31	0.35	0.35
III	1	0	0.14	0	0.32	1	0.71	1	1	1	0.12	0.16	0.29	0.03
IV	0	1	0	0.13	1	0.32	1	0.71	1	1	0.15	0.11	0.03	0.29
V	0.16	0.17	1	1	1	1	0.43	0.43	1	1	0.64	0.63	0.66	0.68
VI	1	1	0	0	0	0	1	1	1	1	0.07	0.09	0.09	0.09

Figure 3: **CLAW partitioned into zones.** (A) ... (B) Details of the zones. From left to right, after zone labels: probability of activation for dSPN, iSPN, GPi, GPeP, Th, GPeA, and STN for left (-L) and right (-R) channels, when networks are in each zone. The grey rows correspond to the launching zone, and the green and blue rows correspond to the zones containing the outer CLAW and inner CLAW states, respectively.

As explicitly mentioned in the Methods section, the details of the procedure used to generate models of the CBGT network are described in two preprints and a paper freely available. However, as these models are central to the argument of the present paper, I think it could benefit from a few more details, clarifying for example the structure of inputs received by the network and the source of variability from trial to trial.

Response: We thank the referee for this helpful suggestion. We have added more details of the model in the Methods section, as follows:

> ... We considered two groups of neurons for each population, corresponding to the left and right action channels, except for the CxI and FSI, which are shared across both channels. At the start of each decision trial, a constant stimulus input to the cortical neurons in both channels was turned on. This input resulted in a nonlinear increase in cortical firing rates and therefore the ramping dynamics across the striatal populations, which impacted downstream activity in the rest of the basal ganglia and thalamic neurons. A choice was declared to be made when the firing rate of the thalamic population within one action channel reached 30 Hz before the firing rate of the other thalamic population. Each neuron evolves according to a conductance-based integrate-and-fire-or-burst model. Individual neurons within the same population were connected to each other with a population-specific probability (p) and connection strength (or weight, w). The maximal synaptic conductance (g) for an existing connection (e.g., AMPA, NMDA, GABA) was given by $g = w$, while it was given by $g = pw$ in a population-averaged

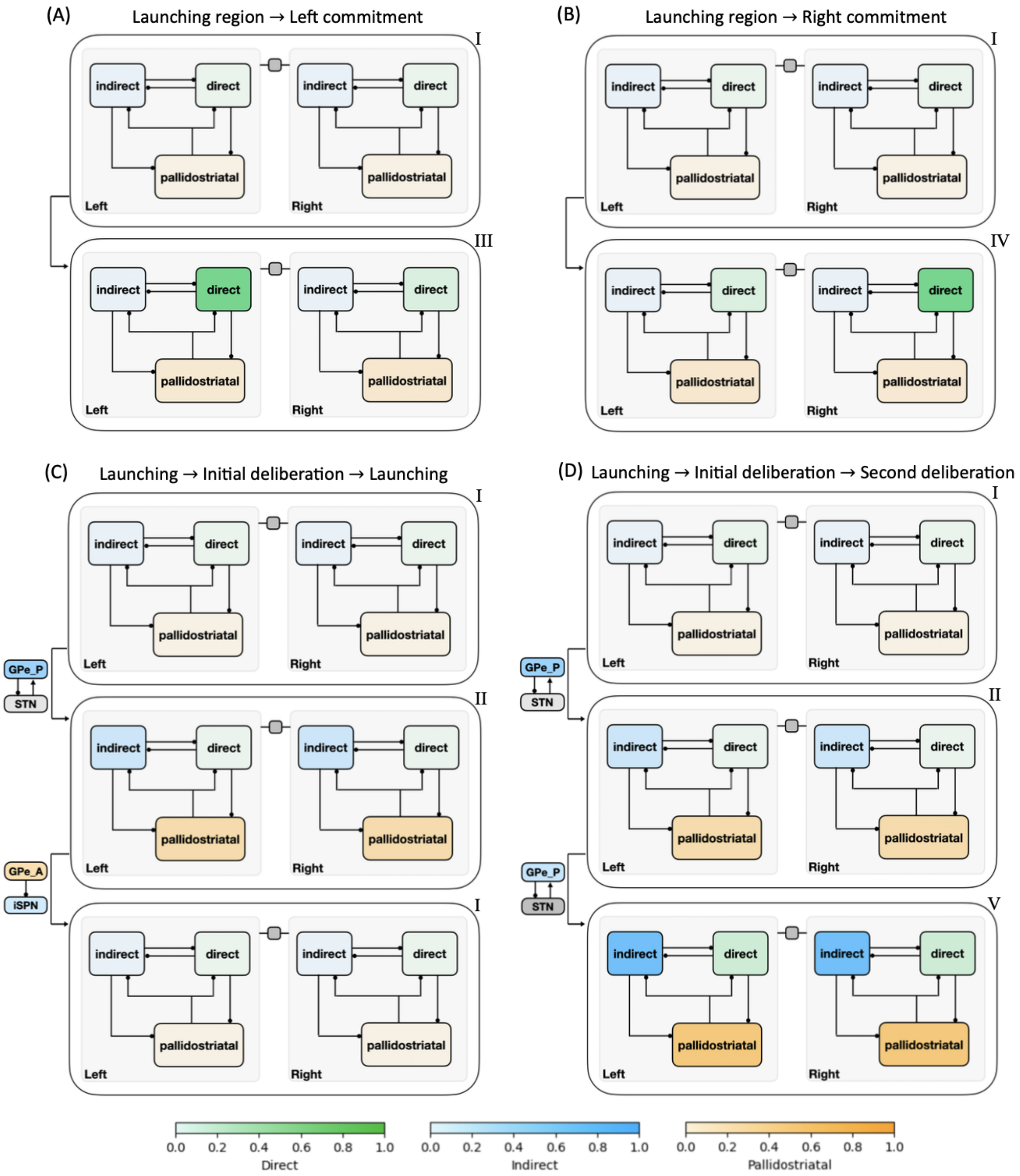

Figure 4: **Temporal activation of CBGT pathways for different decision trajectories along the CLAW.** The direct, indirect, and pallidostriatal pathways across both left and right channels are enclosed in boxes, with their activation strength colored in blue, green, and gold, respectively (color bars at bottom). Darker shades indicate stronger activation within each pathway. Nuclei that play a key role for some phase transitions are indicated near the corresponding arrows. (A) Launching region → Left commitment. (B) Launching region → Right commitment. (C) Launching region → Initial deliberation → Launching region. (D) Launching region → Initial deliberation → Second deliberation. The Roman numeral on the upper right corner of each pattern corresponds to the zone labels in Fig 4.

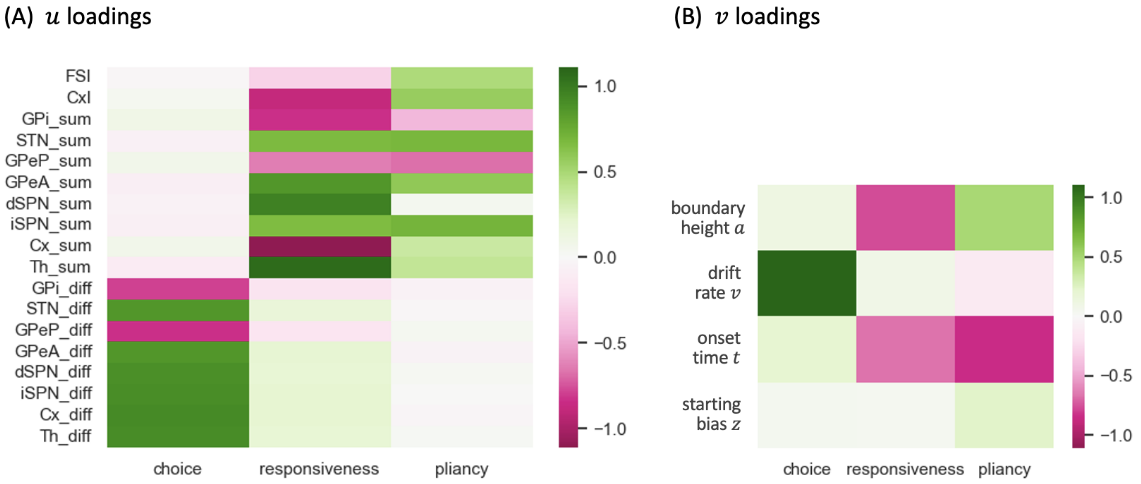

Figure 5: **Canonical loading matrices obtained by applying the CCA.** (A) ... (B) Loading of v, corresponding to DDM parameters. These three components are referred to as choice, responsiveness, and pliancy control ensembles, respectively.

sense. Connections between different populations were similarly characterized by probabilities and weights. These probabilistic connections introduced trial-to-trial variability even with the same CBGT network parameter settings. Figure 1A illustrates the connectivity of the network, and the complete model details can be found in Clapp et al. (2025).

References

Bahuguna, J., Verstynen, T., and Rubin, J. E. (2024). How cortico-basal ganglia-thalamic subnetworks can shift decision policies to maximize reward rate. *bioRxiv*.

Bogacz, R., Brown, E., Moehlis, J., Holmes, P., and Cohen, J. D. (2006). The physics of optimal decision making: a formal analysis of models of performance in two-alternative forced-choice tasks. *Psychological review*, 113(4):700.

Chittka, L., Skorupski, P., and Raine, N. E. (2009). Speed–accuracy tradeoffs in animal decision making. *Trends in ecology & evolution*, 24(7):400–407.

Cisek, P., Puskas, G. A., and El-Murr, S. (2009). Decisions in changing conditions: the urgency-gating model. *Journal of Neuroscience*, 29(37):11560–11571.

Clapp, M., Bahuguna, J., Giossi, C., Rubin, J. E., Verstynen, T., and Vich, C. (2025). Cbgtpy: An extensible cortico-basal ganglia-thalamic framework for modeling biological decision making. *PloS one*, 20(1):e0310367.

Cohen, J. D., McClure, S. M., and Yu, A. J. (2007). Should i stay or should i go? how the human brain manages the trade-off between exploitation and exploration. *Philosophical Transactions of the Royal Society B: Biological Sciences*, 362(1481):933–942.

Dodson, P. D., Larvin, J. T., Duffell, J. M., Garas, F. N., Doig, N. M., Kessaris, N., Duguid, I. C., Bogacz, R., Butt, S. J., and Magill, P. J. (2015). Distinct developmental origins manifest in the specialized encoding of movement by adult neurons of the external globus pallidus. *Neuron*, 86(2):501–513.

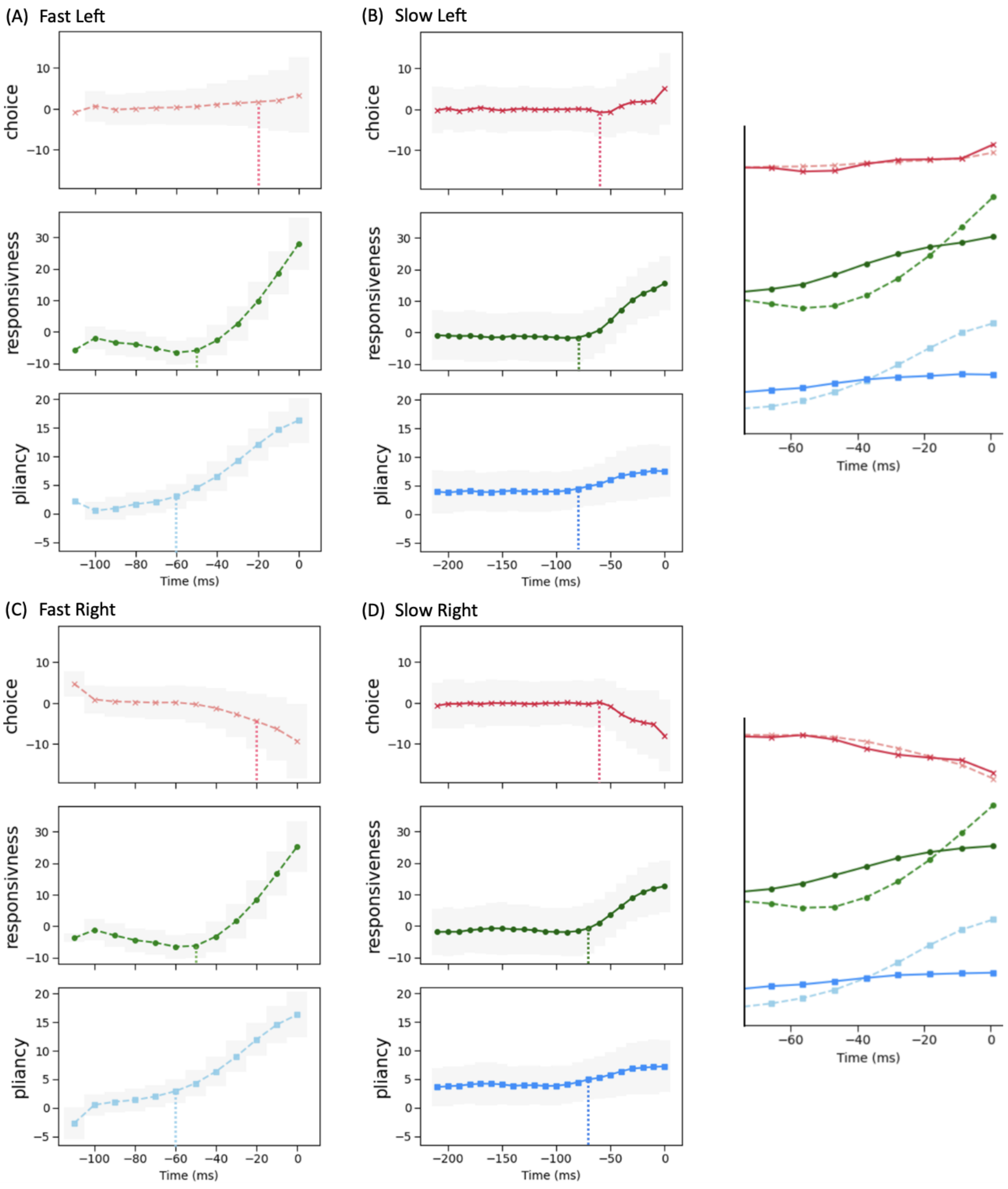

Figure 6: **Time evolution of control ensemble drives.** The percentage changes in each drive are averaged across all trials within each of four decision groups — (A) fast left, (B) slow left, (C) fast right, and (D) slow right, from the stimulus onset time to the decision time (set to be 0). Since each trial had a different decision time, we performed the averaging by aligning trials on their decision times, with the averages represented by dots connected by lines (light dashed for fast and dark solid for slow). Shadowed areas represent the standard deviation for each group. The vertical dotted lines with colors matching the plotted curves mark the timing at which each control ensemble's drive shows a notable response. For fast and slow trials to the same choice, each inset at the right aligns the drives, zoomed in on the time range with pronounced changes (approximately 75 ms), on the same scale for comparison.

Drugowitsch, J., Moreno-Bote, R., Churchland, A. K., Shadlen, M. N., and Pouget, A. (2012). The cost of accumulating evidence in perceptual decision making. *Journal of Neuroscience*, 32(11):3612–3628.

Dunovan, K., Vich, C., Clapp, M., Verstynen, T., and Rubin, J. (2019). Reward-driven changes in striatal pathway competition shape evidence evaluation in decision-making. *PLoS computational biology*, 15(5):e1006998.

Ging-Jehli, N. R., Cavanagh, J. F., Ahn, M., Segar, D. J., Asaad, W. F., and Frank, M. J. (2025). Basal ganglia components have distinct computational roles in decision-making dynamics under conflict and uncertainty. *PLoS biology*, 23(1):e3002978.

Holmes, W. R. and Trueblood, J. S. (2018). Bayesian analysis of the piecewise diffusion decision model. *Behavior research methods*, 50:730–743.

Huang, Y. and Rao, R. P. (2013). Reward optimization in the primate brain: A probabilistic model of decision making under uncertainty. *PloS one*, 8(1):e53344.

Krajbich, I., Armel, C., and Rangel, A. (2010). Visual fixations and the computation and comparison of value in simple choice. *Nature neuroscience*, 13(10):1292–1298.

Malhotra, G., Leslie, D. S., Ludwig, C. J., and Bogacz, R. (2018). Time-varying decision boundaries: insights from optimality analysis. *Psychonomic bulletin & review*, 25:971–996.

Mehlhorn, K., Newell, B. R., Todd, P. M., Lee, M. D., Morgan, K., Braithwaite, V. A., Hausmann, D., Fiedler, K., and Gonzalez, C. (2015). Unpacking the exploration–exploitation tradeoff: A synthesis of human and animal literatures. *Decision*, 2(3):191.

Moran, R. (2015). Optimal decision making in heterogeneous and biased environments. *Psychonomic bulletin & review*, 22:38–53.

Palestro, J. J., Weichart, E., Sederberg, P. B., and Turner, B. M. (2018). Some task demands induce collapsing bounds: Evidence from a behavioral analysis. *Psychonomic bulletin & review*, 25:1225–1248.

Ratcliff, R. (1978). A theory of memory retrieval. *Psychological review*, 85(2):59.

Ratcliff, R. and McKoon, G. (2008). The diffusion decision model: theory and data for two-choice decision tasks. *Neural computation*, 20(4):873–922.

Schouten, J. and Bekker, J. (1967). Reaction time and accuracy. *Acta psychologica*, 27:143–153.

Srivastava, V., Feng, S. F., Cohen, J. D., Leonard, N. E., and Shenhav, A. (2017). A martingale analysis of first passage times of time-dependent wiener diffusion models. *Journal of mathematical psychology*, 77:94–110.

Steinmetz, N. A., Zatka-Haas, P., Carandini, M., and Harris, K. D. (2019). Distributed coding of choice, action and engagement across the mouse brain. *Nature*, 576(7786):266–273.

Thura, D., Cos, I., Trung, J., and Cisek, P. (2014). Context-dependent urgency influences speed–accuracy trade-offs in decision-making and movement execution. *Journal of Neuroscience*, 34(49):16442–16454.

Vich, C., Clapp, M., Rubin, J. E., and Verstynen, T. (2022). Identifying control ensembles for information processing within the cortico-basal ganglia-thalamic circuit. *PLOS Computational Biology*, 18(6):e1010255.

Wyart, V., De Gardelle, V., Scholl, J., and Summerfield, C. (2012). Rhythmic fluctuations in evidence accumulation during decision making in the human brain. *Neuron*, 76(4):847–858.
