# Supplementary material for: How the dynamic interplay of cortico-basal ganglia-thalamic pathways shapes the time course of deliberation and commitment
Source: PLoS Comput Biol. 2026 Mar 9;22(3):e1012966. doi: 10.1371/journal.pcbi.1012966 (PMC12995308; doi:10.1371/journal.pcbi.1012966)
Supplement: Attachment — Submitted filename: rebuttal_auresp_2.pdf [file pcbi.1012966.s007.pdf]

We thank the reviewers for their continued engagement with our work and for their careful reading and thoughtful critiques of our submission. Below, we provide a point-by-point discussion, focusing on the insightful new comments from Reviewer #2 (*italic text*). As you can see, we have revised the manuscript in light of their suggestions. In the manuscript, we have marked new and revised text in red font.

1 Reviewer #1

I would like to thank the Authors for addressing my suggestion so thoroughly.

Response: We thank the reviewer for the supportive comment.

2 Reviewer #2

*I thank the authors for addressing my previous comments, and especially for clarifying the interpretation of some of their results. The motivation for focusing on dynamic DDM parameters is very clear, particularly in light of the new revisions. However, I remain concerned about the methodology used to link CBGT activity to these *dynamic* DDM parameters. In my first review, I wrote: "Although some parameters [. . .] could be interpreted as varying over time, doing so alters their original meaning, which was used to define the decision policy dimensions in the first place". Let me clarify this point: my concern is that correlating CBGT network activity averaged over a trial with loadings of *static* DDM parameters does not ensure that similar relationships hold for near-instantaneous activity (Fig. 7, lines 388-408) or differences in activity between states (Figs. 8-9, lines 469-487) when these are related to *dynamic* DDM parameters. Thus, the *static* loadings might not be usable to infer network drive for *dynamic* decision policies. I think this assumption could be tested. For example, one could run a simple toy-model simulation in which a set of dynamic DDM parameters and their corresponding static parameters are generated. Cross-correlation analyses could then be performed separately:*

- *between trial-averaged activity and static parameters*
- *between within-trial differences in activity and dynamic parameters*

*If the resulting loadings are similar, it would support the use of *static* parameter loadings to infer drives for *dynamic* decision policies. If such a simulation is impractical for reasons I have overlooked, I recommend explicitly noting in the Discussion that some caution is warranted when interpreting this part of the Results. Apart from this point, I have no reservations regarding the rest of the manuscript, and I reiterate my view that it presents highly valuable findings and interesting methodology.*

Response: We appreciate the reviewer's thoughtful concern regarding our use of a static neural–policy mapping to infer dynamic policy changes. We acknowledge that this method rests on an implicit assumption of a shared, stationary mapping, namely that the low-dimensional mapping learned from trial-averaged data remains fixed at the within-trial (or zone-transition) scale that we analyze.

We have implemented the reviewer's proposed toy model comparison (proposal details sent previously via email), but we found that directly comparing the static CCA loadings to the "dynamic CCA on differences"

loadings is not a well-posed comparison. The key issue is that the two CCAs are performed on fundamentally different covariance structures:

- **Raw (trial-averaged) data versus differences reflect distinct sources of variance.** Trial-averaged neural activity is dominated by between-network baseline differences, whereas zone-to-zone or within-trial differences remove baseline offsets and emphasize within-network temporal modulation. As a result, the canonical directions that maximize correlation in the static setting may differ from those obtained from difference data, even when the underlying neural-policy mapping is stationary.

- **Differencing removes time-invariant factors, making loadings non-comparable by construction.** In the reviewer's proposed setup (and in our CBGT/DDM usage), some DDM parameters (e.g., starting bias and onset time) are time-invariant within a trial. Hence, their within-trial differences (Δ) are always zero, and a CCA on Δ-quantities will assign them a zero loading. By contrast, a static CCA on trial-averaged data can assign nonzero loadings to the same parameters because they vary across networks. Therefore, comparing dynamic loadings from Δ-CCA with static loadings (or checking whether the former falls within the static confidence interval) can falsely suggest a mismatch.

- **CCA components are not uniquely defined across fits.** CCA returns multiple canonical components, and when canonical correlations are similar across components, component ordering and loadings can rotate and/or mix across bootstrap resamples. As a result, comparing the loadings of component n across two CCA fits is not rotation-invariant and can be misleading.

For these reasons, we believe that the suggested loading comparison is not the most appropriate test of the underlying assumptions of our approach. However, the central idea, testing whether static axes generalize to dynamic inference, is exactly the right question. To address this point, we performed a generalization and alignment test that directly targets this question, and we have added the following details about this test to the Supporting Information S2 Appendix, as well as some clarifications in the main text.

> **S2 Appendix. Validation of static-to-dynamic neural–policy mapping**. To evaluate whether static CCA loadings learned from trial-averaged data (Fig 6) can support inference about time-resolved (dynamic) DDM parameters (Figs 7–9), we implemented a toy model simulation in which neural population activity $\mathbf{f}(t) \in \mathbb{R}^N$ was generated from DDM variables $\mathbf{d}(t) \in \mathbb{R}^4$ according to
>
> $$\mathbf{f}(t) = W(t)\mathbf{d}(t) + \epsilon(t),$$
>
> where $\epsilon(t)$ denotes Gaussian white noise. We considered two regimes of neural-policy mapping. **(A) Stationary mapping**: $W(t) \equiv W$, where the same linear mapping applied across time and across networks. **(B) Non-stationary mapping**, where the mapping $W(t)$ changed piecewise over time. For regime B, we further distinguished two cases: **(B1)** $W(t)$ **shared across networks** and **(B2)** $W(t)$ **varying across networks**. The core idea was to fit a static CCA using trial-averaged data from a training subset of networks, yielding canonical loading pairs $(\mathbf{u}_i, \mathbf{v}_i)$. Then for held-out networks, we applied the learned canonical axes at three temporal resolutions: (i) trial-averaged (sanity check), (ii) zone/bin differences ($\geq$10 ms; the scale relevant to Figs 7–9), and (iii) moment-to-moment differences ($\leq$1 ms; the hardest case). For example, for zone-to-zone transitions, the resulting neural firing and decision policy canonical scores were respectively given by
>
> $$s_{i,k,z}^{(f)} = \mathbf{u}_i^\top \Delta\mathbf{f}_{k,z}, \qquad s_{i,k,z}^{(d)} = \mathbf{v}_i^\top \Delta\mathbf{d}_{k,z},$$
>
> where i, k, and z index the canonical component, network, and zone, respectively. We then assessed their alignment using a rotation-invariant metric: for each canonical dimension i, we

computed the Pearson correlation between neural firing and decision policy canonical scores across held-out networks, and summarized performance by the best-aligned canonical dimension, i.e.,

$$\mathcal{A}(z) = \max_i \left| \mathrm{corr}_k \big(s^{(f)}_{i,k,z},\ s^{(d)}_{i,k,z} \big) \right| .$$

This metric directly tests whether the static neural-policy axes generalize to dynamic changes.

The results of this analysis are shown in panel A of the Supporting Information S2 Figure (or see Fig. 1A below). In the left subpanel (trial-averaged activity), alignment was near-maximal under both the stationary mapping (blue) and the shared dynamic mapping (green), indicating that the static CCA axes were robust in these regimes. In contrast, alignment was low under the network-varying dynamic mapping (red), reflecting that no single low-dimensional mapping could generalize across networks when the mapping itself was network-specific. In the middle subpanel (zone/bin differences), alignment remained high for the stationary mapping and for the shared dynamic mapping with coarse temporal scale but decreased substantially at finer scale and collapsed when mappings varied across networks. Hence, the static CCA loadings could still generalize to dynamic fluctuations only when the underlying neural–policy relationship was shared across networks and varied moderately over time. In the right subpanel (moment-to-moment differences), alignment decreased further even under stationary and shared dynamic mappings, consistent with the increasing dominance of transient fluctuations. From this toy model, we clarify that using static CCA loadings to infer dynamic DDM parameters is justified under the regime where the underlying neural-policy mapping is stationary or shared across networks and slowly time-varying, but not when the mapping becomes network-specific. In the former regime, the static axes are sufficiently stable to remain informative at the temporal scale of interest (e.g., zone/bin dynamics) but not necessarily at the moment-to-moment level.

This leads to the key question for our CBGT simulation: which of these regimes did the underlying neural–policy mapping fall into? As a first step toward applying the toy-model generalization logic to our simulated CBGT data, we assessed the reliability of the static CCA mapping itself. Specifically, using network-level bootstrap resampling of the 300 simulated CBGT networks, we repeatedly refit the static CCA and examined the resulting distributions of canonical correlations (panel B of S2 Figure) and loading structures (panel C). The canonical correlations were highly reproducible across bootstrap replicates: the choice component correlation remained near maximal, the responsiveness component was similarly stable, and the pliancy component was somewhat less consistent but still strongly correlated, indicating that the overall strength of the static neural–policy relationship is reliable. We then evaluated whether each individual variable preserves its loading structure between CCA components across refits. For each neural variable and each DDM parameter, we quantified its component-loading profile by taking the squared CCA loadings across the three components (making the measure sign-invariant) and normalizing them within each variable to sum to one, yielding the fraction of that variable's loading magnitude assigned to each component. Because CCA components can vary across bootstrap refits, we compared profiles in an order-insensitive manner by sorting these three fractions within each variable. Loading-structure stability was then quantified as a bounded similarity score based on the L^1 distance between the bootstrap and full-data profiles (1 = identical profile), and summarized across bootstrap resamples for each variable (panel C). We observed that the DDM parameters showed tightly preserved loading structure across resamples (median stability > 0.9). Neural variables showed more variability but were nonetheless also quite stable (median stability ≥ 0.8). This pattern reflects the fact that in our CBGT simulations, multiple neural populations contribute at comparable levels (Fig 6A), so small changes in the fitted CCA basis can shift how a given population's loading is partitioned across the

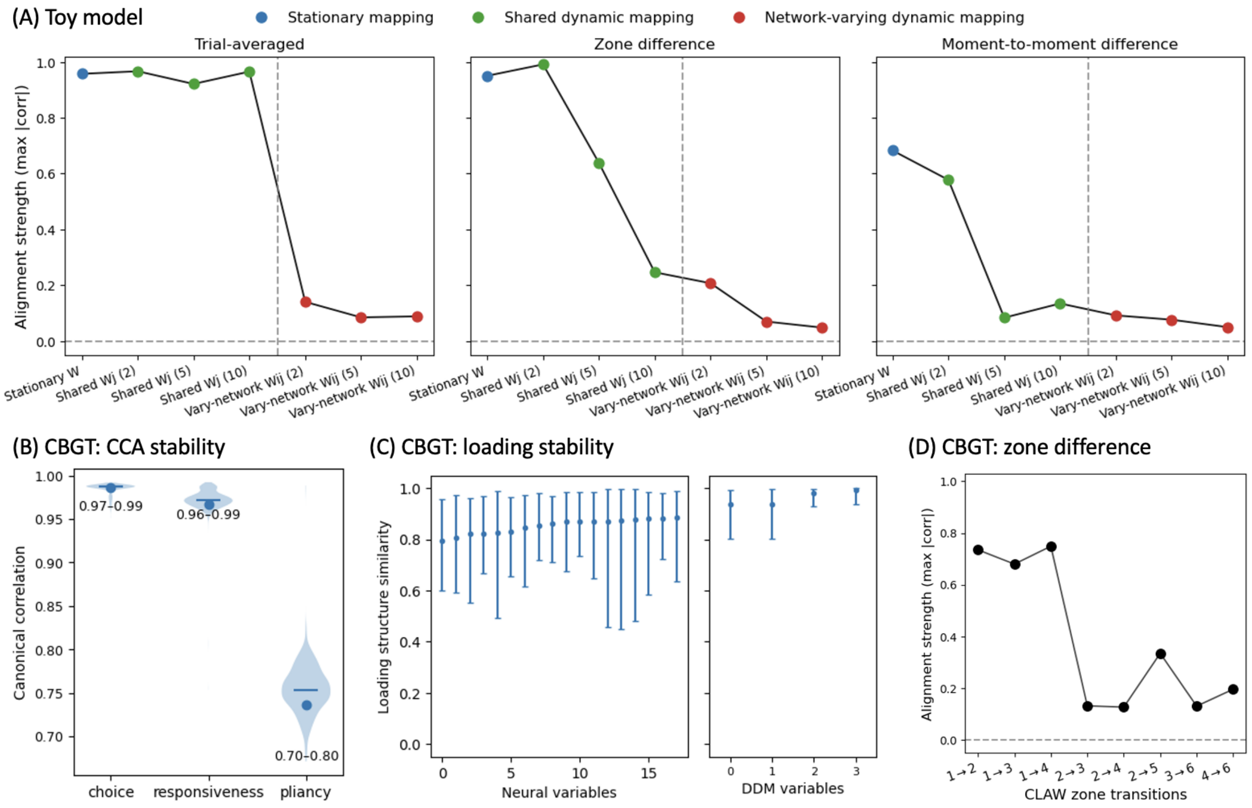

Figure 1: **Generalization of static neural–policy mappings across temporal scales and mapping regimes.** (A) Toy model. Alignment strength (maximum absolute correlation across canonical dimensions) between neural projections and DDM parameter projections is shown for three temporal resolutions: trial-averaged activity (left), zone/bin differences (middle), and moment-to-moment differences (right). Results are reported for three classes of generative models: a stationary mapping (blue), a shared dynamic mapping that changed across 2, 5, or 10 time segments but was shared across networks (green), and a network-varying dynamic mapping (red). Static CCA axes were learned from trial-averaged data and evaluated on held-out networks. Alignment remained high under stationary and shared dynamic mappings at coarse temporal resolution, degraded with finer resolution, and collapsed when mappings varied across networks. (B) Static CCA was refit on bootstrap resamples of networks from the simulated CBGT data. Left: bootstrap distributions of canonical correlations for the three CCA components, with dots marking full-data estimates and horizontal lines marking bootstrap medians. Right: bootstrap distributions of Spearman rank correlations quantifying the stability of variable-importance rankings on the neural and DDM sides. Numbers denote 95% bootstrap intervals. (B) CBGT: bootstrap stability of canonical correlations. Static CCA was refit on network-level bootstrap resamples of the simulated CBGT dataset. Violin plots show bootstrap distributions of canonical correlations for the three CCA components, with dots marking full-data estimates and horizontal lines marking bootstrap medians; numbers denote 95% bootstrap intervals. (C) CBGT: bootstrap stability of loading structures. For each variable, we compared its loading profile across CCA components (sign- and order-insensitive) between bootstrap refits and the full-data fit; points and error bars show per-variable similarity (95% bootstrap intervals) for neural (left) and DDM (right) variables. (D) CBGT: zone difference. To estimate the dynamic alignment in simulated CBGT data, we used the same train/test approach and alignment metric as in the toy model. Static CCA axes learned from trial-averaged data were applied to CLAW zone-to-zone CBGT activity differences.

three components. Importantly, these shifts occur within the same low-dimensional subspace, rather than reflecting a different mapping across bootstrap refits. Together, the tight canonical-correlation intervals and stable per-variable loading structures support the existence of a shared low-dimensional neural–policy mapping across networks and argue against a network-specific regime (illustrated by the red case in the left subpanel of panel A), in which both the correlation and loading structure would be expected to fluctuate markedly across bootstrap fits.

Having established that the static CCA in our CBGT simulation is reliably estimated and shared across networks, we finally applied the same train/test generalization approach and alignment metric used in the toy model to the CLAW zone-to-zone differences. However, in the CBGT simulations, DDM parameters were available only as static per-network fits derived from choices and decision times, so time-resolved DDM trajectories were not available and could not be used to serve as ground truth. Nevertheless, the toy model shows that the primary issue for validating static-to-dynamic inference is whether the neural–policy mapping $W(t)$ is shared or varies across networks, and we therefore tested a weaker, mapping-focused condition. The neural activity in zone or time bin z can be expressed as

$$\mathbf{f}_k(z) = W(z)\mathbf{d}_k^{\text{static}} + \mathbf{b}_k(z),$$

where $\mathbf{b}_k$ captures zone/bin-dependent effects not explained by the static DDM parameters (e.g., baseline offsets, latent network-specific state, or other unmodeled dynamics). Taking differences yields

$$\Delta\mathbf{f}_{k,z_1\to z_2} = (W(z_2) - W(z_1))\mathbf{d}_k^{\text{static}} + \Delta\mathbf{b}_{k,z_1\to z_2}.$$

Under this formulation, we can apply static loadings learned from trial-averaged data of training networks to held-out networks by computing neural and policy scores,

$$s_{i,k,z}^{(f)} = \mathbf{u}_i^\top \Delta\mathbf{f}_{k,z_1\to z_2}, \qquad s_{i,k,z}^{(d)} = \mathbf{v}_i^\top \mathbf{d}_k^{\text{static}}$$

and evaluating their alignment strength $\mathcal{A}(z)$.

Applying this analysis to the simulated CBGT data (panel D of S2 Figure), we found that alignment was high for early CLAW zone transitions and decreased for later transitions. This graded pattern again rules out a network-specific mapping (cf. panel A, red), which would yield uniformly weak alignment, and it also rules out a strictly stationary mapping (cf. panel A, blue), which would yield uniformly high alignment. Instead, the observed pattern falls in the regime of a shared but phase-dependent mapping (cf. panel A, green). In the CBGT simulations, early zones (e.g., launching and initial deliberation) spanned a larger proportion of the decision process and therefore more closely reflected the trial-averaged neural–policy relationship captured by the static CCA axes, leading to stronger alignment. By contrast, later zones (e.g., commitment, reversal, and second deliberation) were more strongly shaped by transient fluctuations, i.e., larger contributions from the $\Delta\mathbf{b}_{k,z}$ term, that weakened the alignment. Note that the alignment strength in the middle subpanel of A was computed by pooling across all zone/bin transitions, whereas here we plot the alignment separately for each CLAW zone transition. On average, the overall alignment here was large and positive across all zone transitions, indicating that the underlying mapping did not fall into the network-varying regime, identified in the toy model analysis, in which static axes are invalid for dynamic inference. Thus, the above analysis supports our claim that in our CBGT simulations, the static CCA loadings provide a valid low-dimensional subspace for inferring drives for dynamic decision policies where the temporal scale of interest was relatively coarse ($\geq$10 ms), even though they are not expected to capture moment-to-moment ($\leq$1 ms) fluctuations.

And, we have made the following clarifications in the main text.

- In the Methods section where this mapping is discussed (at the end of Control ensembles):

> ... Finally, we projected W_{ij} to the DDM parameter space via $P_{ij} = W_{ij}V^T$ where the v loadings comprise the columns of V, yielding a p by 4 matrix. The median of each column of P_{ij} reflects the overall change in the corresponding DDM parameter from zone i to zone j, as shown in Fig 8. Because our zone-resolved DDM analysis relied on the static CCA mapping, we evaluated the validity of this static-to-dynamic inference using generalization and alignment tests (see Supporting Information S2 Appendix).

- In the Results where this mapping is mentioned:

> ... we finally projected W_{ij} to the DDM space. Using the components of the v loadings to form the columns of V, we computed $P_{ij} = W_{ij}V^T \in \mathbb{R}^{p\times 4}$ which relates the firing rate changes to changes in the DDM parameters. Hence, the median of all p projections in each column of P_{ij} is proportional to the overall change in the direction and magnitude of the corresponding decision policy parameter in the transition from zone i to zone j. We also evaluated the extent to which the static CCA loadings remain informative for time-varying decision policy dynamics using a generalization-and-alignment test; the full analysis is reported in the Supporting Information S2 Appendix.

- In the Discussion, where we mentioned translating changes in CBGT network activity associated with transitions through CLAW zones into evolution of DDM parameters, we added the following text to the end of this paragraph:

> ... Hence, the time-varying framework that we have derived serves as a more complete representation of of how decision-making dynamics can be flexibly tuned to prioritize either speed or accuracy across diverse scenarios. One caveat is that our analysis used a static CCA mapping to infer time-varying DDM parameter dynamics. This step has been shown mathematically justifiable in our simulations at coarse temporal resolution (e.g., $\geq$ 10 ms bins), but becomes less accurate at very fine, near-instantaneous timescales (e.g., $\leq$ 1 ms) (see Supporting Information S2 Appendix and S2 Figure), so our conclusions are more speculative in those cases.

Minor points: Line 590: "mulitple"; Line 654: "of of"

Response: We thank the referee for pointing these out, and we have corrected the typos.
